# Prospective serological and molecular cross-sectional study focusing on *Bartonella* and other blood-borne organisms in cats from Catalonia (Spain)

**DOI:** 10.1186/s13071-021-05105-6

**Published:** 2022-01-04

**Authors:** Alejandra Álvarez-Fernández, Ricardo Maggi, Gerard Eduard Martín-Valls, Marta Baxarias, Edward Bealmear Breitschwerdt, Laia Solano-Gallego

**Affiliations:** 1 Department of Animal Medicine and Surgery, Facultat de Veterinària, Universitat Autònoma de Barcelona, Cerdanyola del Valles, Spain; 2 Department of Clinical Sciences and the Intracellular Pathogens Research Laboratory, Comparative Medicine Institute, College of Veterinary Medicine, North Carolina State University (NCSU), Raleigh, NC USA; 3 Department of Animal Health and Anatomy, Facultat de Veterinària, Universitat Autònoma de Barcelona, Cerdanyola del Valles, Spain

**Keywords:** Bartonellosis, Hemotropic *Mycoplasma*, *Mycoplasma wenyonii*, *Ehrlichia*, *Anaplasma*, Piroplasma, Co-infection, Cats, Spain

## Abstract

**Background:**

There is limited clinical or epidemiological knowledge regarding *Bartonella* infection in cats, and no serological studies have compared the presence of antibodies against different *Bartonella* species. Moreover, there are limited feline *Bartonella* studies investigating co-infections with other vector-borne pathogens and the associated risk factors. Therefore, the objective of this study was to investigate *Bartonella* spp. infections and co-infections with other pathogens in cats from Barcelona (Spain) based on serological and/or molecular techniques and to determine associated risk factors.

**Methods:**

We studied colony and owned cats (*n* = 135). Sera were tested for *Bartonella henselae*-, *Bartonella quintana*-, and *Bartonella koehlerae*-specific antibodies using endpoint in-house immunofluorescence antibody assays. *Bartonella* real-time PCR (qPCR) and conventional PCR (cPCR) were performed. In addition, cPCR followed by DNA sequencing was performed for other pathogenic organisms (*Anaplasma*, *Babesia*, *Cytauxzoon*, *Ehrlichia*, *Hepatozoon*, hemotropic *Mycoplasma*, and *Theileria* spp.).

**Results:**

From 135 cats studied, 80.7% were seroreactive against at least one *Bartonella* species. *Bartonella quintana*, *B. koehlerae*, and *B. henselae* seroreactivity was 67.4, 77.0, and 80.7%, respectively. Substantial to almost perfect serological agreement was found between the three *Bartonella* species. Colony cats were more likely to be *Bartonella* spp.-seroreactive than owned cats. Moreover, cats aged ≤ 2 years were more likely to be *Bartonella* spp.-seroreactive. *Bartonella* spp. DNA was detected in the blood of 11.9% (*n* = 16) of cats. Cats were infected with *B. henselae* (*n* = 12), *B. clarridgeiae* (*n* = 3), and *B. koehlerae* (*n* = 1). *Mycoplasma* spp. DNA was amplified from 14% (*n* = 19) of cat blood specimens. Cats were infected with *Mycoplasma haemofelis* (*n* = 8), *Candidatus* M. haemominutum (*n* = 6), *Candidatus* Mycoplasma turicensis (*n* = 4), and *Mycoplasma wenyonii* (*n* = 1). *Anaplasma*, *Babesia*, *Cytauxzoon*, *Ehrlichia* spp., *Hepatozoon*, and *Theileria* spp. DNA was not amplified from any blood sample. Of the 16 *Bartonella* spp.-infected cats based on PCR results, six (37%) were co-infected with *Mycoplasma* spp.

**Conclusions:**

*Bartonella* spp. and hemoplasma infections are prevalent in cats from the Barcelona area, whereas infection with *Anaplasma* spp., *Babesia*, *Cytauxzoon*, *Ehrlichia* spp., *Hepatozoon*, and *Theileria* infections were not detected. Co-infection with hemotropic *Mycoplasma* appears to be common in *Bartonella*-infected cats. To our knowledge, this study is the first to document *M. wenyonii* is infection in cats.

**Graphical Abstract:**

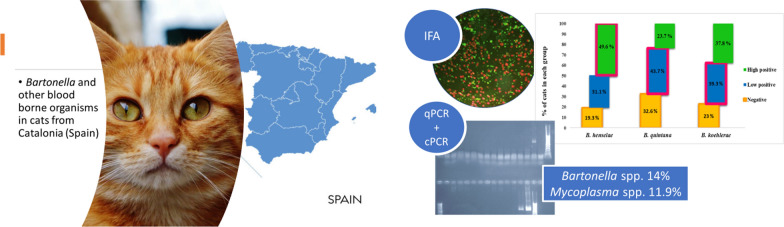

## Background

Bartonellosis, caused by *Bartonella* spp. parasites, is a vector-borne infectious disease that is currently considered an emerging zoonosis [1]. More than 40 *Bartonella* species that are adapted to infect a broad spectrum of reservoir mammalian hosts, including cats, are described in the literature [2, 3]. Transmission to cats is mainly by flea feces, potentially ticks, and scratches and bites between hosts. The cat has been described as the main reservoir for *Bartonella henselae*, *Bartonella clarridgeiae* and *Bartonella koehlerae* [4]. However, cats can be sporadically infected with two other zoonotic *Bartonella* species: *Bartonella quintana* [5, 6] and *Bartonella vinsonii* subsp. *berkhoffii* [7].

The spectrum of disease manifestations associated with *Bartonella* spp. infections in cats continues to expand [8], despite the fact that it is not easy to demonstrate an association between clinical signs, laboratory abnormalities, and *Bartonella* spp. infection [9, 10]. This factor is primarily due to the long duration of relapsing bacteremia and the high percentage of infected healthy cats in endemic areas [3, 11]. Although the majority of acute infections caused by *Bartonella* spp. are thought to be self-limiting in cats [12], persistent infections can be associated with a wide variety of clinical signs and abnormalities. These manifestations in cats can range from intra- or extra-erythrocytic subclinical bacteremia to fever of unknown origin, lymphadenomegaly, endocarditis, myocarditis, ocular disease (neuroretinitis, uveitis), skin inflammation, and other less common disease manifestations [13, 14]. Various factors allow *Bartonella* spp. to persist in the blood of hosts, causing a chronic intravascular and endotheliotropic infection that can ultimately result in the appearance of nonspecific or specific clinical manifestations. Factors that influence symptomatology include virulence differences among *Bartonella* spp. and strains, the mode of transmission, differences in the host immune response and clinical status (comorbidities), concurrent infectious or noninfectious diseases, bacterial load, therapeutic- or infection-induced immunosuppression, and malnutrition [15, 16].

Due to the abovementioned factors, establishing disease causation or a diagnosis of *Bartonella* spp. infections can be clinically challenging, particularly in cats. There are no available diagnostic techniques whose negative result guarantees the absence of infection [3]. Under this premise, infection can be confirmed only by positive diagnostic test results derived from molecular modalities, such as conventional (cPCR) or real-time PCR (qPCR), ideally accompanied by DNA sequencing, or the isolation and identification of the bacteria by enrichment culture, rather than exposure [17, 18]. In addition to technical limitations inherent in culture and PCR diagnostic techniques, *Bartonella* may not be present in sufficient quantities in the blood at the time of specimen collection to be detected. As an example, *Bartonella* DNA was amplified from fresh frozen tissues of dogs with hemangiosarcoma, where qPCR from blood failed to amplify bacterial DNA [19]. Thus, choosing the correct sample for culture or PCR testing could also be critical for the definitive diagnosis of bartonellosis [19].

Indirect immunofluorescence assays (IFA) are the most frequently used serological technique for the detection of antibodies against *Bartonella* spp. [20–23], but other serological assays are available, such as enzyme-linked immunosorbent assay (ELISA) and western immunoblot [12, 24]. A serological negative result does not ensure that a cat is not infected with a *Bartonella* sp., and a positive result only documents the presence of antibodies against the pathogen, but does not confirm infection. ‬‬‬‬Although technically challenging and more expensive than selecting individual tests, considering a combination of diagnostic techniques and optimal specimen types in conjunction with the correct interpretation of the results is likely a good strategy to increase ‬‬‬‬‬‬‬‬‬‬‬‬‬‬‬‬‬‬‬‬‬‬‬‬‬‬‬‬‬‬‬‬‬‬ diagnostic success in cats with suspected bartonellosis [3, 19].‬‬‬‬‬‬‬‬‬‬

*Bartonella* spp. epidemiological studies involving cats, carried out in many parts of the world, have documented substantial differences in prevalence from one area to another [3, 25]. In Europe, many studies including various cat populations have reported seroprevalence ranging from 0 to 71.4% and molecular prevalence rates ranging from 0 to 83.5% [3, 26, 27]. In Europe, including Spain, clinical and epidemiological knowledge regarding *Bartonella* infection in cats remains limited. Furthermore, there are no serological studies comparing the presence of antibodies against different *Bartonella* species in cats, and only a few molecular studies have assayed bacteremia in cats [28, 29]. In addition, there are limited feline *Bartonella* studies that investigated co-infections with other vector-borne pathogens and the associated risk factors [29, 30]. The objective of this study was to test cat serum samples for the presence of antibodies against *B. henselae*, *B. quintana*, and *B. koehlerae* antigens. To assess for co-infections, blood samples were tested by PCR for *Anaplasma* spp., *Bartonella* spp., *Ehrlichia* spp., and hemotropic *Mycoplasma* spp. and using primers that amplified *Babesia*, *Cytauxzoon*, *Hepatozoon*, and *Theileria* spp.

## Methods

A prospective cross-sectional study, conducted between 2017 and 2019 in cats from Barcelona province (Spain), was designed to investigate *Bartonella* spp. seroprevalence and bacteremia prevalence using qPCR and cPCR, with all DNA amplicons sequenced to confirm species identity. In addition, co-infection with other feline vector-borne pathogens was assessed by cPCR testing.

### Cats and specimen collection

A total of 117 blood specimens from apparently healthy cats and 18 blood specimens from sick cats were collected by venipuncture. A general physical examination was performed in all cats. A clinical questionnaire was completed for each cat including information about age, sex, colony origin or owned pet, breed, weight, clinical history, travel history, health status (sick versus healthy), exposure to fleas and/or ticks or bites, and the use of acaricide products. In most cases (*n* = 110), the blood specimens were obtained under anesthesia during a neutering procedure. A signed consent form was obtained from the owners or by the colony origin person in charge of the cats.

Approximately 8–10 ml of peripheral blood was collected by jugular or cephalic venipuncture from each cat at the time of enrollment. Two milliliters was collected into an EDTA-anticoagulant tube for DNA extraction, and 4–6 ml was injected into serum separator tubes with clot accelerator and granule serum separator for serology. Samples were subsequently stored at −80 °C until testing.

### *Bartonella* IFA serological testing

For all 135 cats, three IFAs were used to detect serum antibodies directed against three *Bartonella* species (*B. henselae*, *B. quintana*, and *B. koehlerae* antigens) as described previously [31, 32]. Briefly, to obtain antigens for IFA testing, *B. henselae* SA2+ (feline origin Missy S 95 FO-099), *B. quintana* cynomolgus monkey origin 11-MO-01 (China/Pfizer), and *B. koehlerae* (originally from blood of *Trillium B*, feline) were passed from agar-grown cultures of each organism into DH82 (a continuous canine histiocytic cell line) cultures. For each antigen, infected cell cultures were spotted onto 30-well Teflon-coated slides (Cel-Line/Thermo Scientific), air-dried, acetone-fixed, and stored frozen. Serum samples were diluted in phosphate-buffered saline (PBS) solution containing 1% normal goat serum, 0.05% Tween 20, and 0.5% powdered nonfat dry milk (Bio-Rad, Hercules, CA, USA) to block nonspecific antigen binding sites. Cat sera were further tested with twofold dilutions out to a final dilution of 1:16384, and 10 µl of each serum dilution was applied per well. Previously tested positive and negative controls were selected and added in each slide. The slides were incubated for 30 min at 37 °C and then washed with PBS under moderate agitation for an additional 30 min. Once slides were dry, 10 µl of fluorescein-conjugated goat anti-cat immunoglobulin G (IgG) (MP Biomedicals) at a dilution of 1:100 was added into each well. The slides were incubated for another 30 min at 37 °C in the dark to protect the photosensitive conjugate. The washing procedure described above was repeated, adding a few drops of Tween 20 (Sigma-Aldrich). After the last washing procedure, some drops of antifade mounting medium (Vectashield, Vector Labs, Burlingame, CA, USA) were added on the cover slips. The slides were evaluated using a fluorescence microscope (Leica DM6000 B; Leica Microsystems, Wetzlar, Germany) at ×200 and ×400 magnification, and each well was compared to the fluorescence pattern seen in the positive and negative controls. To avoid confusion with possible nonspecific binding found at low dilutions, a cutoff of 1:64 was selected as a seroreactive antibody titer [31, 32]. Antibody titer results were classified as low seroreactivity from 1:64 to 1:512 and high seroreactivity > 1:512 for comparative analyses [32].

### Blood DNA purification

EDTA tubes were centrifuged at 1300×*g* for 5 min. Plasma was obtained for further studies and the cellular pellet was used for DNA extraction. Total DNA was extracted from blood cell pellets in 110 samples and from EDTA whole blood in 25 samples using the DNA Gene extraction kit (Sigma-Aldrich) following the manufacturer’s instructions, with slight modifications. Forty microliters of proteinase K solution was added to all samples. Four hundred microliters of whole blood was used for all sample DNA extractions. The other steps were performed as described previously [33]. Distilled water was used as a negative control for all DNA extractions.

### Conventional and quantitative real-time PCR analysis

DNA extracted from each blood sample was screened for the presence of *Bartonella* spp. using cPCR and qPCR, and for *Ehrlichia* spp., *Anaplasma* spp., piroplasmids, and hemotropic *Mycoplasma* spp. DNA using only cPCR. The primers used for cPCR and qPCR to establish species strain identification by amplicon product size or melting temperature, respectively, are summarized in Table 1.

**Table 1:** Target gene employed, size in base pairs of amplicons and cPCR and qPCR primer sequences used

Target gene	Size in base pairs (bp)	Primer names and sequences	References
Conventional PCR			
* Bartonella* spp. 16S-23S ITS	500–680 bp	BsppITS325s: 5′ CTTCAGATGATGATCCCAAGCCTTTGGCG 3′ BsppITS1100as: 5′ GAACCGACGACCCCCTGCTTGCAAAGCA 3′	[19]
* Mycoplasma* spp. (hemotropic group) 16S rRNA	588 bp	Myco16S-322 s 5′ GCCCATATTCCTACGGGAAGCAGCAGT 3′ Myco16S-938as 5′ CTCCACCACTTGTTCAGGTCCCCGTC 3′	[19]
* Anaplasma* or *Ehrlichia* 16S rRNA	420 bp	GEPs 5′ CTGGCGGCAAGCYTAACACATGCAAGTCGAACGGA 3′ GEPr 5′ CTTCTTCTRTRGGTACCGTCATTATCTTCCCYAYTG 3′	[33]
* Babesia*, *Cytauxzoon*, *Hepatozoon*, and *Theileria* spp. 18S rRNA	663 bp	Piro18S-144 s 5′ ACCGTGCTAATTGTAGGGCTAATACA 3′ Piroplasma18S-722as 5′ GAATGCCCCCAACCGTTCCTATTAAC 3′	[19]
Real-time PCR			
* Bartonella* spp. 16S-23S ITS	200–231 bp	BsppITS325s: 5′ CTTCAGATGATGATCCCAAGCCTTYTGGCG 3′ BsppITS543as: 5′ TAAAYTGGTGGGCCTGGGAGGACTTG 3′ Probe BsppITS500: 5′ FAM-GTTAGAGCGCGCGC TTGATAAG—IABkFQ 3′	[34]

#### Conventional PCR analysis

Amplification was performed in a 25-µl final volume reaction containing 12.5 µl of Taq-Ex^®^ Premix (Fisher Scientific), 0.2 µl of 100 µM of each forward and reverse primer (Roche Diagnostics, Basel, Switzerland) 7.1 µl of distilled H_2_O, and 5 µl of DNA from each sample tested. PCR negative controls were prepared by adding distilled H_2_O. Positive controls were used in each reaction and were prepared by serial dilution (using cat blood DNA) of genomic DNA from each pathogen down to 0.001 pg/μl (equivalent to 0.5 bacteria/μl). Conventional PCR was performed in a VeritiPro Thermal Cycler (Applied Biosystems, Waltham, MA, USA) under the following conditions: a single hot-start cycle at 95 °C for 2 min followed by 55 cycles of denaturing at 94 °C for 15 s, annealing at 66 °C for 15 s, and extension at 72 °C for 18 s. Amplification was completed by an additional cycle at 72 °C for 1 min, and products were analyzed by 1.5% agarose gel electrophoresis with detection using ethidium bromide under ultraviolet light.

#### Real-time PCR analysis

*Bartonella* spp. qPCR was performed using a 7500 Fast Dx Real-Time PCR instrument (Applied Biosystems™) in 25-µl final volume reactions. Master mix was prepared using 12.5 µl of buffer and 1 µl of enzyme mix (AgPath-ID™ One-Step RT-PCR Reagents, Applied Biosystems™), 5.9 µl of H2O, 0.2 µl of 100 µM of fluorescent probe, and 0.2 µl of 100 µM of each primer (Roche Diagnostics). PCR testing was carried out using 5 µl of DNA. PCR negative and positive controls were prepared as described for cPCR. Real-time PCR conditions were as follows: a single hot-start cycle at 95 °C for 3 min followed by 44 cycles of denaturing at 94 °C for 10 s, 10 s of annealing at 66 °C, and extension at 72 °C for 10 s. When present, *Bartonella* spp. amplicons were detected by fluorescence readings at the appropriate wavelength [19].

### Sequencing of positive PCR amplicons

The molecular characterization of *Bartonella* and hemotropic *Mycoplasma* spp., as well as the confirmation of cPCR and qPCR positive results, was performed by Sanger sequencing of cPCR and qPCR amplicons, followed by chromatographic evaluation and sequence alignment. For bacterial species identification, DNA sequences were analyzed by comparing similarity with other sequenced bacteria deposited in the GenBank database using the Basic Local Alignment Search Tool (BLAST). Only PCR results confirmed by sequencing were considered positive in this study.

### Statistical analysis

A descriptive analysis was carried out for the data from each cat along with a comparative analysis according to the IFA and PCR results. Frequency analysis was performed for age, sex, colony/owned, ectoparasites, health status, and three *Bartonella* spp. IFA, cPCR, and qPCR results. Comparative analysis of categorical data was performed using the Chi-square test or Fisher’s exact test. The kappa (κ) statistic was used to assess the degree of (inter-rater) agreement between IFAs performed against three *Bartonella* species. The Shapiro–Wilk test was performed to detect the normality of the distribution of the samples. A *P*-value < 0.05 was considered statistically significant. Statistical analyses were performed using the R i386 version 3.3.1 (R Development Core Team) and the DeduceR version 1.7-16 (DeduceR: A data Analysis GUI for R) software programs for Windows.

## Results

### Cat description

All cats sampled were European domestic shorthairs. Seventy-five of 135 cats (55.5%) were female and 60 (44.5%) were male. Only one female and two males were intact. All remaining cats were neutered. Cat age ranged from 4 months to 7 years, with a median age of 1.2 years. Cats ≤ 2 years old were considered young cats and > 2 years old were considered old cats. Fifty-eight percent (79/135) were colony cats and 41.5% (56/135) were owned pet cats. A recent history of tick exposure or flea infestation was reported in 81.5% (110/135), whereas 18.5% (25/135) were flea- or tick-infested at the time of blood collection, including 23 colony cats and two owned pet cats. Colony cats were more likely to have a recent history of ectoparasites (100%) compared to owned cats (3.6%) (Fisher’s exact test: *P* < 0.001, OR = 3466.2, 95% confidence interval [CI] = 163.2–73,624.6). Routine treatment with ectoparasiticides was reported for 18.5% (25/135) of the cats. One hundred and seventeen cats (86.7%) were clinically healthy, and 18 (13.3%) cats had clinical signs at the time of sampling. Gingivitis was the most frequent clinical sign, found in 55.6% (10 of 18, 95% CI = 32.6–78.5%) of the sick cats).

### *Bartonella* IFA in cat serum samples

**Fig. 1:**
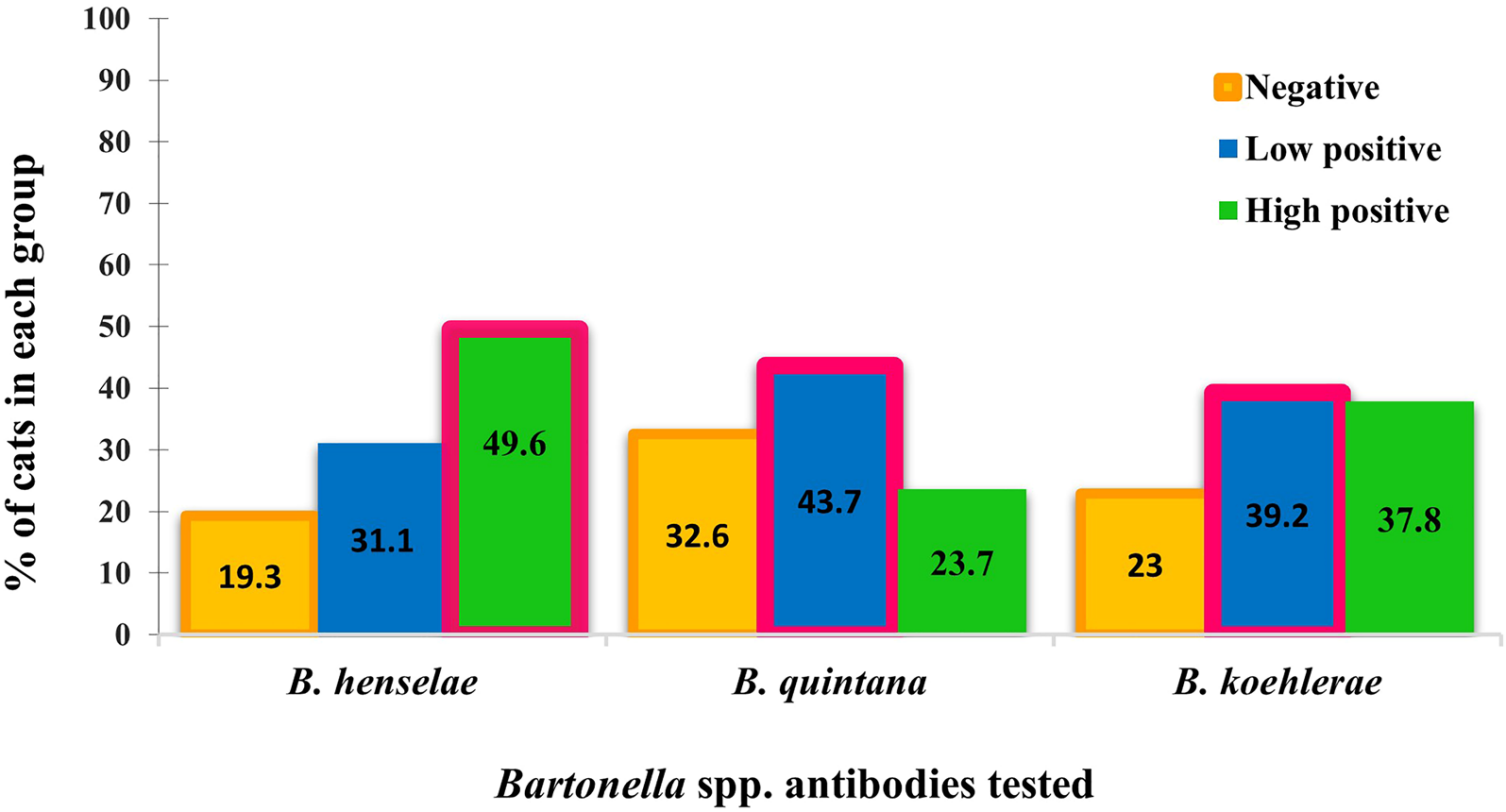
Frequencies of negative, low seroreactive, and high seroreactive by means of IFA for the three *Bartonella* spp. antigens studied. *Bartonella* spp. antibody titers were classified as low seroreactive from 1:64 to 1:512 and high seroreactive > 512. The most prevalent group is boxed in pink for each *Bartonella* sp.

Considering antibody titers ≥ 1:64, 80.7% (109 of 135, 95% CI = 74.1–87.4%) of the cats were seroreactive against at least one *Bartonella* sp. antigen. *Bartonella henselae*, *B. quintana*, and *B. koehlerae* seroreactivity was 80.7% (109 of 135, 95% CI = 74.1–87.4%), 67.4% (91 of 135, 95% CI = 59.5–75.3%), and 77% (104 of 135, 95% CI = 69.9–84.1%), respectively. Eighty-eight of 109 cats (80.7%, 95% CI = 73.3–88.1%) were seroreactive against all three *Bartonella* spp. antigens, 32 cats (29.3%, 95% CI = 20.8–37.9%) were seroreactive against two *Bartonella* species, and five cats (4.6%, 95% CI = 0.7–8.5%) were seroreactive against one *Bartonella* species. Interestingly, all cats that were seroreactive against *B. koehlerae* or *B. quintana* antigens were also seroreactive against *B. henselae* IFA antigens. Five cats had antibodies against *B. henselae* but were not seroreactive to the other two *Bartonella* spp. Kappa agreement analysis between *Bartonella* spp. IFA is summarized in Table 2. Cats seroreactive against *B. quintana* and *B. koehlerae* antigens were more likely to be *B. henselae*-seroreactive (100%) than seronegative (Fisher’s exact test: *P* < 0.001, OR = 262.9, 95% CI = 15.3–4515.9 and OR = 1081.6, 95% CI = 57.3–20,432.6). The serum IFA geometric mean antibody titer for *B. henselae*, *B. quintana*, and *B. koehlerae* antigens was 1:2050, 1:1028, and 1:1460, respectively. The maximum titer was 1:16384 for *B. henselae* and 1:8192 for *B. quintana* and *B. koehlerae*. The frequency of seronegative, low seroreactive, and high seroreactive antibody titers for the three *Bartonella* antigens studied is graphically displayed in Fig. 1.

**Table 2:** Comparison of agreement between IFAs performed against three *Bartonella* species

Test pair	κ ± SE	κ interpretation^a^
*B. henselae* IFA versus *B. quintana* IFA	0.661 ± 0.070	Substantial agreement
*B. henselae* IFA versus *B. koehlerae* IFA	0.889 ± 0.048	Almost perfect agreement
*B. quintana* IFA versus *B. koehlerae* IFA	0.726 ± 0.065	Substantial agreement

^a^The interpretation for each κ value is shown in the final column according to the following scale: ≤ 0, no agreement; 0.01–0.20, none to slight; 0.21–0.40, fair; 0.41–0.60, moderate; 0.61–0.80, substantial; and 0.81–1.00, almost perfect agreement. IFA, immunofluorescence antibody assay; κ, Cohen’s kappa value; SE, standard error

When IFA results were compared with data collected from each cat, we found that colony cats were more likely to be *Bartonella* spp. (100%)-seroreactive than owned pet cats (53%) (Fisher’s exact test: *P* < 0.001, OR = 136.9, 95% CI = 8–2319.8). Cats with a recent history of tick and flea infestation were more likely to be *Bartonella* spp. (99%)-seroreactive (Fisher’s exact test: *P* < 0.001, OR = 5450, 95% CI = 177.8–167,014.7) when compared with non-infested cats. Cats with ectoparasites were more likely to be *B. henselae*-seroreactive (100%) (Fisher’s exact test: *P* = 0.001, OR = 15.5, 95% CI = 0.9–263.2), *B. koehlerae*-seroreactive (100%) (Fisher’s exact test: *P* = 0.004, OR = 19.6, 95% CI = 1.2–332.5), and *B. quintana*-seroreactive (84%) (Fisher’s exact test: *P* = 0.05, OR = 3, 95% CI = 1–9.4). Cats ≤ 2 years old were more likely to be *Bartonella* spp.-seroreactive (91.5%) than cats > 2 years old (41.4%) (Chi-square test: *χ*^2^ = 36.8, *df* = 1, *P* < 0.001). Of the 109 cats that were seroreactive against any *Bartonella* species tested, 17 cats were sick (15.6%, 95% CI = 8.8–22.4%), whereas the remaining cats were clinically healthy. There was no statistical difference associated with serology and health status. No further significant associations were found.

### PCR and sequencing

*Bartonella* spp. DNA was amplified from the blood of 11.9% (16 of 135, 95% CI = 6.4–17.3%) of the cats using cPCR or qPCR. Based on DNA sequence alignments, cats were infected with *B. henselae* (*n* = 12), *B. clarridgeiae* (*n* = 3), or *B. koehlerae* (*n* = 1). From 16 positive cats, *Bartonella* DNA was amplified using cPCR in 10 cases (Table 3) and using qPCR in eight cases (Table 4). *Bartonella* DNA was amplified by both cPCR and qPCR in only two of 16 cats. Based on PCR testing, 81.3% (95% CI = 62.1–100.4%) of bacteremic cats originated from a colony and were ≤ 2 years of age. Of the 16 *Bartonella* spp.-infected PCR-positive cats, 15 were *Bartonella* spp.-seroreactive. Fourteen out of 15 *Bartonella*-seroreactive and PCR-positive cats had IFA antibody titers ≥ 1:1024 to one or more test antigens.

**Table 3:** *Bartonella* spp. 16S-23S internal transcribed spacer sequences obtained by cPCR (primers Bspp. 325–1100) compared with sequences available in the GenBank database by nucleotide sequence homology using BLAST

ID	GenBank accession no.	*Bartonella* spp.	Homology % (bp)	Compared sequence GenBank accession no.
GUAB-14	OK624784	*B. henselae*	100% (409/409)	JQ316963.1
GUAB-27	OK624785	*B. henselae*	99.45% (364/366)	EF209013.1
GUAB-35	OK624786	*B. henselae*	100% (409/409)	JQ316963.1
GUAB-59	OK624787	*B. clarridgeiae*	99.77% (429/430)	AB896695.1
GUAB-60	OK624788	*B. henselae*	99.96% (585/587)	DQ529247.1
GUAB-75	OK624789	*B. henselae*	100% (458/458)	KY464065.1
GUAB-77	OK624790	*B. henselae*	100% (393/393)	HM042285.1
GUAB-78	OK624791	*B. clarridgeiae*	100% (525/525)	AB896695.1
GUAB-79	OK624792	*B. henselae*	100% (365/365)	DQ383228.1
GUAB-105	OK624793	*B. clarridgeiae*	100% (509/509)	MN170544.1

*ID* identification, % percentage, *bp* base pairs

**Table 4:** *Bartonella* spp. 16S-23S internal transcribed spacer sequences obtained by qPCR (primers Bspp 325–543) compared with sequences available in the GenBank database by nucleotide sequence homology using BLAST

ID	GenBank accession no.	Species	Homology % (bp)	Compared sequence GenBank accession no.
GUAB-14	OK624794	*B. henselae*	99% (148/149)	AB896699.1
GUAB-35	OK624795	*B. henselae*	100% (140/140)	MT095053.1
GUAB-39	OK624796	*B. henselae*	100% (148/148)	AB896699.1
GUAB-41	OK624797	*B. henselae*	99% (167/168)	MT095054.1
GUAB-45	OK624798	*B. henselae*	100% (151/151)	MT095050.1
GUAB-46	OK624799	*B. henselae*	99% (148/149)	MT095048.1
GUAB-49	OK624800	*B. henselae*	100% (144/144)	MT095053.1
GC-13	OK624801	*B. koehlerae*	94% (120/127)	KX499345.1

*ID* identification, % percentage, *bp* base pairs

Hemotropic *Mycoplasma* spp. DNA was amplified by cPCR from 14% (19/135, 95% CI = 8.2–19.9%) of the cats. Based on DNA sequencing, *Mycoplasma haemofelis* (*n* = 8), *Candidatus* M. haemominutum (*n* = 6), *Candidatus* M. turicensis (*n* = 4), and *Mycoplasma wenyonii* (*n* = 1) were identified. *Anaplasma* spp., *Babesia*, *Cytauxzoon*, *Ehrlichia* spp., *Hepatozoon*, and *Theileria* spp. were not detected in any of the samples studied.

For all DNA amplified by PCR, sequences were compared with sequences available in the GenBank database by nucleotide sequence homology using BLAST. Percentage of similarity, species, sequence description, and Genbank accession number are summarized in Tables 3, 4, and 5.

**Table 5:** Hemotropic *Mycoplasma spp.* 16S-23S internal transcribed spacer sequences obtained by cPCR compared with sequences available in the GenBank database by nucleotide sequence homology using BLAST

ID	GenBank accession no.	Hemotropic *Mycoplasma* spp.	Homology % (bp)	Compared sequence GenBank accession no.
G-UAB-27	OK624802	*M. haemofelis*	93% (466/499)	MN240855.1
G-UAB-30	OK624803	*M. haemofelis*	99% (508/509)	EU442638.1
G-UAB-37	OK624804	*M. haemofelis*	100% (526/526)	KU645929.1
G-UAB-40	OK624805	*Candidatus* M. haemominutum	95% (533/560)	KR905456.1
G-UAB-48	OK624806	*M. haemofelis*	95% (496/524)	MG594502.1
G-UAB-49	OK624807	*Candidatus* M. haemominutum	100% (547/547)	MT926039.1
G-UAB-58	OK624808	*Candidatus* M. haemominutum	100% (508/508)	MN240865.1
G-UAB-60	OK624809	*M. haemofelis*	99% (529/530)	KU645929.1
G-UAB-63	OK624810	*Candidatus* M. haemominutum	99% (471/472)	KM275257.1
G-UAB-67	OK624811	*Candidatus* M. turicensis	97% (499/512)	KY046312.1
G-UAB-70	OK624812	*M. haemofelis*	100% (515/515)	KU645929.1
G-UAB-77	OK624813	*M. wenyonii*	99% (539/546)	MT241310.1
G-UAB-78	OK624814	*Candidatus* M. turicensis	100% (480/480)	MK632342.1
G-UAB-79	OK624815	*Candidatus* M. turicensis	99% (461/466)	MK632342.1
G-UAB-88	OK624816	*Candidatus* M. haemominutum	100% (534/534)	MT926039.1
G-UAB-94	OK624817	*Candidatus* M. haemominutum	100% (546/546)	KR905457.1
G-UAB-109	OK624818	*M. haemofelis*	100% (31/31)	MK632350.1
G-UAB-112	OK624819	*M. haemofelis*	99% (529/530)	KU645929.1
G-UAB-115	OK624820	*Candidatus* M. turicensis	99% (467/468)	EU789558.1

*ID* identification, % percentage, *bp* base pairs

Based upon PCR results, 29 of 135 cats (21.5%, 95% CI = 14.6–28.4%) were infected with at least one bacterial organism. Seven cats (5.2%, 95%; CI = 1.4–8.9%) were co-infected with two pathogens based on molecular assays. *Bartonella* spp.-infected PCR-positive cats were more likely to be infected with hemotropic *Mycoplasma* spp. (37.5%) than *Bartonella* spp. PCR-negative cats (10.9%) (Fisher’s exact test: *P* = 0.0115, OR = 4.89, 95% CI = 1.5–15.7).

Detailed clinical, serological, and PCR data for infected cats is summarized in Table 6. Two cats had gingivitis and one a purulent nasal discharge. There were no statistical associations when hemotropic *Mycoplasma* PCR results were compared with categorical clinical data or *Bartonella* spp. IFA serology results.

**Table 6:** Bacteremic PCR-positive cats and the corresponding IFA results for each of the three *Bartonella* spp. IFA antigens

Cat ID	Lifestyle	Age (months)	Clinical signs	Ectoparasites	IFA			cPCR *Bartonella*	qPCR *Bartonella*	cPCR hemotropic *Mycoplasma*
					*B. henselae*	*B. quintana*	*B. koehlerae*			
GUAB-14	O	24	AH	Np	1:1024	1:256	1:1024	*B. henselae*	*B. henselae*	Neg
GUAB-27	C	8	AH	Np	1:512	1:512	1:512	*B. henselae*	Neg	*M. haemofelis*
GUAB-35	C	6	Gingivitis	Np	1:512	1:4096	1:2048	*B. henselae*	*B. henselae*	Neg
GUAB-39	C	18	AH	*Otodectes cynotis*	1:4096	1:1024	1:4096	Neg	*B. henselae*	Neg
GUAB-41	C	6	AH	Np	1:1024	1:4096	1:1024	Neg	*B. henselae*	Neg
GUAB-45	C	24	Purulent nasal discharge	Fleas	1:4096	1:2048	1:2048	Neg	*B. henselae*	Neg
GUAB-46	C	18	AH	Np	1:4096	1:1024	1:1024	Neg	*B. henselae*	Neg
GUAB-49	C	8	AH	Np	1:4096	1:2048	1:2048	Neg	*B. henselae*	*Candidatus* M. haemominutum
GUAB-59	C	24	AH	Fleas	1:2048	1:256	1:512	*B. clarridgeiae*	Neg	Neg
GUAB-60	C	36	AH	Fleas	1:2048	1:1024	1:2048	*B. henselae*	Neg	*M. haemofelis*
GUAB-75	C	5	AH	Np	1:1024	1:1024	1:8192	*B. henselae*	Neg	Neg
GUAB-77	C	6	AH	Np	1:2048	1:1024	1:2048	*B. henselae*	Neg	*M. wenyonii*
GUAB-78	C	6	AH	Np	1:8192	1:1024	1:2048	*B. clarridgeiae*	Neg	*Candidatus* M. turicensis
GUAB-79	C	6	AH	Np	1:8192	1:256	1:2048	*B. henselae*	Neg	*Candidatus* M. turicensis
GUAB-105	C	10	Gingivitis	Fleas	1:4096	1:512	1:512	*B. clarridgeiae*	Neg	Neg
GC-13	O	38	AH	Np	Neg	Neg	Neg	Neg	*B. koehlerae*	Neg

*O* owned, *C* colony, *AH* apparently healthy, *Np* not present, *Neg* Negative, *cPCR* conventional polymerase chain reaction, *qPCR* real-time polymerase chain reaction

## Discussion

The *Bartonella* spp. seroprevalence in this study was 80.7%. Similar seroprevalence results (71.4%) were reported in a study published in 2006 involving cats from the Barcelona area [31]. There has seemingly been little progress in preventing flea exposure among colony/stray and owned pet cats in this region. Despite the high *Bartonella* spp. seroprevalence, 84.4% of the seroreactive cats were apparently healthy. Gingivitis was the most frequent clinical finding among the sick cats. In addition, clinical abnormalities observed in *Bartonella* spp.-infected cats in this study were mild, highlighting the historically long and apparently efficient co-evolution of fleas, cats, and *Bartonella* spp., as well as the poor relationship between *Bartonella* seroreactivity and clinical status. In the present study, seroprevalence in owned and colony cats was 53% and 100%, respectively. However, other studies performed in the Barcelona area found seroprevalence of 29.6% in owned pet cats [36] and 44% in shelter or colony cats when cats in both studies were tested only with a *B. henselae* assay [28]. Seroprevalence reported in cats from other areas of Spain has ranged from 24.7% in the Madrid area [29] to 50% described in a study that included several locations within the Mediterranean areas of Spain (Canary Islands, Galicia, and Madrid) [37].

To the best of our knowledge, this is the first study measuring antibodies against three different species of *Bartonella* and the first evidence of *B. quintana* and *B. koehlerae* seroreactivity in cats from Spain. In this study, 49.6% of seroreactive cats had high antibody titers against *B. henselae* (≥ 1:1024), compared with a previous study from the same area carried out 17 years earlier that reported 23% seroreactivity, with maximum antibody titers of 1:512 [37]. Interestingly, the geometric mean antibody titer in the present study was 1:2050, compared to 1:256 in the previous study, which may reflect age-related differences in seroreactivity [31]. The mean age of cats in the present study and in the previous study was 1.2 and 4.4 years, respectively, suggesting that young cats may have higher antibody titers than older cats. In Europe, similar seroprevalence has been reported in Portugal (64.9%) against *Bartonella* spp. [38] and in Germany (68.7%) against *B. henselae* antigen [39]. Variations between studies are likely due to differences in the study populations (e.g., owned versus colony) and differences in *Bartonella* spp. prevalence among geographic areas, differences in flea exposure, and differences in diagnostic techniques used among published studies [29, 40].

In this study, all *Bartonella* spp.-seroreactive cats had previously been exposed to fleas and/or ticks, in agreement with the well-known relationship between exposure to fleas and the transmission of *Bartonella* spp. infection in cats [41–44]. Although *Bartonella* transmission by ticks remains controversial, there is enough evidence (i.e., artificial membrane feeding system experiments, clinical and epidemiological studies in cats, dogs, and humans) to consider that *Ixodes ricinus* ticks can act as a vector for *B. henselae* [45–48]. The presence of *Bartonella* spp. DNA has also been documented in *Rhipicephalus sanguineus* ticks removed from cats and dogs [44, 49, 50]. However, less information is available regarding the potential role of *R. sanguineus* ticks or other tick species as potential vectors for *Bartonella* spp. transmission to cats, dogs, or humans [44, 51].

Several studies have assessed serological cross-reactivity between *Bartonella* species or strains in cats [52, 53], dogs [54], and humans [55, 56]. In our study, 65.2% of the cats examined had antibodies against all three *Bartonella* spp. IFA antigens. All cats that were seroreactive against *B. koehlerae* or *B. quintana* antigens were also seroreactive against *B. henselae* antigen. In addition, substantial to almost perfect agreement was found between the three species of *Bartonella* studied. Furthermore, the three cats infected with *B. clarridgeiae* had high antibody titers against *B. henselae* (1:2048–1:8192), *B. quintana* (1:256–1:1024), and *B. koehlerae* (1:512–1:2048). The present findings suggest serological cross-reactivity, as previously reported in cats [49, 50], or that cats were co-infected with several *Bartonella* spp. when infested with numerous fleas or both fleas and ticks. In this study, increasing the number of antigens by adding *B. quintana* and *B. koehlerae* to *B. henselae* did not increase the number of seroreactive cats. When experimentally infected with *B. henselae* or *B. vinsonii* subsp. *berkhoffii*, dogs naturally infected with *B. koehlerae* elicited a species-specific antibody response for the first 100 days of the study [57]. Differentiating cross-reactivity from co-exposures to multiple *Bartonella* spp., potentially occurring at multiple vector exposure time points among naturally exposed animals, often infested with numerous fleas and ticks, remains difficult to impossible with current diagnostic modalities.

The majority of young cats ≤ 2 years were *Bartonella* spp.-seroreactive. Furthermore, 14 of 16 PCR-seroreactive cats were ≤ 2 years of age, although compared to cats older than 2 years, there was no significant difference. In agreement with previous studies, young cats were more likely to be bacteremic and seroreactive [58, 59]. Moreover, in the present study, all cats with *Bartonella* spp. antibody titers ranging from 1:512 to 1:8192 were bacteremic, which is consistent with a previous study [58] and suggests that antibodies do not elicit effective immune elimination of bacteremia. ‬‬‬‬‬‬‬‬‬‬‬‬‬‬‬‬‬‬‬‬‬‬‬‬‬‬‬‬‬‬‬‬‬‬‬‬‬‬‬‬‬‬‬‬‬‬‬‬‬‬‬‬‬‬‬‬‬‬‬‬‬‬‬‬‬‬‬‬‬‬‬‬‬‬‬‬‬‬‬‬‬‬‬‬‬‬‬‬‬‬‬‬‬‬‬‬‬‬‬‬‬‬‬‬‬‬‬‬‬‬‬‬‬‬‬‬‬‬‬‬‬‬‬‬‬‬‬‬‬‬‬‬‬‬‬‬‬‬‬‬‬‬‬‬‬‬‬‬‬‬‬‬‬‬‬‬‬‬‬‬‬‬‬‬‬‬‬‬‬‬‬‬‬‬‬‬‬‬‬‬‬‬‬‬‬‬‬‬‬‬‬‬‬‬‬‬‬‬‬‬‬‬‬‬‬‬‬‬‬‬‬‬‬‬‬‬‬‬‬‬‬‬‬‬‬‬‬‬‬‬‬‬‬‬‬‬‬ ‬‬‬‬‬‬‬‬‬‬‬‬‬‬‬‬‬‬‬‬‬‬‬‬‬‬‬‬‬‬‬‬‬‬‬‬‬‬‬‬‬‬‬‬‬‬‬‬‬‬‬‬‬‬‬‬‬‬‬‬‬‬‬‬‬‬‬‬‬‬‬‬‬‬‬‬‬‬‬‬‬‬‬‬‬‬‬‬‬‬‬‬‬‬‬‬‬‬‬‬‬‬‬‬‬‬‬‬‬‬‬‬‬‬‬‬‬‬‬‬‬‬‬‬‬‬‬‬‬‬‬‬‬‬‬‬‬‬‬‬‬‬‬‬‬‬‬‬‬‬‬‬‬‬‬‬‬‬‬‬‬‬‬‬‬‬‬‬‬‬‬‬‬‬‬‬‬‬‬‬‬‬‬‬‬‬‬‬‬‬‬‬‬‬‬‬‬‬‬‬‬‬‬‬‬‬‬‬‬‬‬‬‬‬‬‬‬‬‬‬‬‬‬‬‬‬‬‬‬‬‬‬‬‬‬‬‬‬‬‬‬‬‬‬‬‬‬‬‬‬‬‬‬‬‬‬‬‬‬‬‬‬‬‬‬‬‬‬‬‬‬‬‬‬‬‬‬‬‬‬‬‬‬‬‬‬‬‬‬‬‬‬‬‬‬‬‬‬‬‬‬‬‬‬‬‬‬‬‬‬‬‬‬‬‬‬‬‬‬‬‬‬‬‬‬‬‬‬‬‬‬‬‬‬‬‬‬‬‬‬‬‬‬‬‬‬‬‬‬‬‬‬‬‬‬‬‬‬‬‬‬‬‬‬‬‬‬‬‬‬‬‬‬‬‬‬‬‬‬‬‬‬‬‬‬‬‬‬‬‬‬‬‬‬‬‬‬‬‬‬‬‬‬‬‬‬‬‬‬‬‬‬‬‬‬‬‬‬‬‬‬‬‬‬‬‬‬‬‬‬‬‬‬‬‬‬‬‬‬‬‬‬‬‬‬‬‬‬‬‬‬‬‬‬‬‬‬‬‬‬‬‬‬‬‬‬‬‬‬‬‬‬‬‬‬‬‬‬‬‬‬‬‬‬‬‬‬‬‬‬‬‬‬‬‬‬‬‬‬‬‬‬‬‬‬‬‬‬‬‬‬‬‬‬‬‬‬‬‬‬‬‬‬‬‬‬‬‬‬‬‬‬‬‬‬‬‬‬‬‬‬‬‬‬‬‬‬‬‬‬‬‬‬‬‬‬‬‬‬‬‬‬‬‬‬‬‬‬‬‬‬‬‬‬‬‬‬‬‬‬‬‬‬‬‬‬‬‬‬‬‬‬‬‬‬‬‬‬‬‬‬‬‬‬‬‬‬‬‬‬‬‬‬‬‬‬‬‬‬‬‬‬‬‬‬‬‬‬‬‬‬‬‬‬‬‬‬‬‬‬‬‬‬‬

*Bartonella* spp. DNA was detected in 11.9% of the cats in this study. The most prevalent *Bartonella* species based on molecular analysis was *B. henselae* (8.9%), also consistent with worldwide PCR data [3], followed by *B. clarridgeiae* (1.5%) and *B. koehlerae* (0.7%). The prevalence for *B. henselae* bacteremia has ranged from 0.3 to 38.3% in different areas of Spain [28, 29, 36, 60, 61]. *Bartonella clarridgeiae* infections have previously been reported in the Barcelona area, with similar bacteremia results ranging from 0.6 to 1% [31, 60]. A higher *B.* *clarridgeiae* bacteremia prevalence (10.9%) was reported in northern Spain [62]. In Europe, bacteremic prevalence rates for various combinations of *B. clarridgeiae*, *B. henselae*, and *B. koehlerae* have ranged from 0.7 to 83.5% [27, 63–65]. The differences in bacteremic prevalence are related to different climatic conditions, the cat population tested, and PCR techniques used, as well as intrinsic differences in bacteremic behavior linked to the microorganisms themselves. For example, it is possible that cats infected with *B. koehlerae* maintain a lower level of bacteremia than cats infected with *B. henselae* or *B. clarridgeiae,* as *B. koehlerae* is infrequently isolated or detected by PCR testing [3, 66]. Despite this low bacteremic prevalence, cats are considered the main reservoir of *B. koehlerae* infection worldwide [67–69]*.*

To the best of our knowledge, this is the first description of *B. koehlerae* infection in an apparently healthy cat from Spain, as has previously been reported in Greece [65], France [70], Israel [71], and the USA [67, 72]. Moreover, *B. koehlerae* infections have also been described in dogs from Israel [73], Spain [35, 74], and the USA [75], and infection has been diagnosed sporadically in humans from Israel [76] and the USA [77, 78]. Interestingly, *B. koehlerae* infection was not diagnosed in a large study in veterinary personnel from Spain, despite 41.6% of participants being *B. koehlerae*-seroreactive, using the same antigen as used in this study [22]. However, there is more worldwide evidence of *B. koehlerae* DNA in cat fleas, with prevalence exceeding 30% [79–83], suggesting that *B. koehlerae* infections in cats are potentially more common than studies have documented thus far [3]. In the present study, the *B. koehlerae* DNA-positive cat was seronegative against all three test antigens. In an experimental study, seroconversion was detected between of 7 to 15 days after *B. koehlerae* inoculation in cats [53]. The cats infected in that study had serological cross-reactivity with *B. henselae* antigens, but antibody titers against *B. henselae* were lower than *B. koehlerae* antibody titers [53]*.* Therefore, we might hypothesize that this cat was recently infected or did not seroconvert despite being chronically infected, a phenomenon commonly described in bacteremic dogs [84]. Furthermore, it is important to highlight that in the present study, *B. koehlerae* was 94.49% identical to the *B. koehlerae* subsp. *bothieri* strain 1178 16S-23S ribosomal RNA intergenic spacer partial sequence as described previously [85], and therefore, this microorganism might be a new *B. koehlerae* strain or a closely related *Bartonella* species. For this reason, differences in antigenicity among *B. koehlerae* strains could make detection of antibodies in infected cats more difficult [85].

Although *B. quintana* seroprevalence was documented in this study, *B. quintana* has not previously been amplified from the blood of any cat in Spain. To the best of our knowledge, there is no previous molecular evidence documenting *B. quintana* infection in cats in Spain; however, *B. quintana* has been previously documented in sick humans [86, 87] and healthy humans in Spain [22, 88]. Nevertheless, *B. quintana* infection was documented from cat dental pulp in France [5] and from two bacteremic feral cats in the USA [6]. Failure to isolate or PCR-amplify *B. quintana* DNA from cats may reflect infrequent or low-level bacteremia, potentially requiring enrichment blood culture/PCR or testing at multiple time points to document potential *B. quintana* infection. Previous research has confirmed the ability of five *Bartonella* species including *B. quintana* to persist in *C. felis* [43], and *B. quintana* DNA has been amplified from cat fleas in France [80]. Therefore, fleas could be a potential alternative vector for *B. quintana*, although flea transmission has not yet been proven.

In the present study, the hemotropic *Mycoplasma* infection rate was 14%, including the amplification and sequencing of *M. haemofelis* (5.9%), *Candidatus* M. haemominutum (4.4%), and *Candidatus* M. turicensis (3%) DNA. Frequency and species distribution are similar and in agreement with previous studies from Spain, where hemotropic *Mycoplasma* prevalence of 7.8% [28] and 12% [69] was reported from the Barcelona area and 10.6% from Madrid [89]. Similar prevalence and species distribution have also been documented in Italy (11.6% and 18.3%) [90, 91] and Ireland (16.4%) [92]. However, higher hemotropic *Mycoplasma* prevalence has been reported from Greece (26.4%) [30] and Portugal (27.1%) [93]. Therefore, hemotropic *Mycoplasma* spp. are widely distributed and prevalent among European cats. Furthermore, co-infection with hemotropic *Mycoplasma* and *Bartonella* spp. appears to be highly common among cats in the Barcelona area. *Bartonella* spp. and hemotropic *Mycoplasma* spp. co-infection prevalence was 4.4% in this study, compared with other cat studies from Italy that reported co-infection prevalence of 3% [91] and 0.1% [90]. However, *Bartonella* and hemotropic *Mycoplasma* co-infection has not been documented in other feline [65] or canine [94] studies. Interestingly, *Bartonella* spp. and hemotropic *Mycoplasma* spp. co-infection has been sporadically reported in humans [95, 96]. It remains unknown whether there are factors that predispose cats or humans to co-infection with *Bartonella* spp. and hemotropic *Mycoplasma* spp.; therefore, further studies are needed to elucidate these findings.

Surprisingly, and to the best of our knowledge, this is the first time that *M. wenyonii* DNA has been amplified from the blood of a cat worldwide and the first description of this hemoplasma in an animal from Spain. *Mycoplasma wenyonii* has been widely identified in bovines worldwide [97–99] but also sporadically documented in other ungulates such as water buffalo and red deer from central Europe [100], France [101], and Cuba [102]. The mode of transmission for the majority of hemotropic *Mycoplasma* species, including *M. wenyonii*, remains unknown [101]. One study reported potential vertical transmission for *M. wenyonii.* in bovines [103]. Interestingly, *M. wenyonii* DNA was confirmed in one wild-caught mosquito pool by DNA sequencing in the USA [104]. Those mosquitos were captured near feral cat colonies to specifically evaluate wild-caught mosquitoes for evidence of hemotropic *Mycoplasma* species DNA and to determine whether the feline hemoplasmas could be transmitted by *Aedes aegypti* mosquitoes in a laboratory setting. Laboratory transmission to naive cats was not documented, suggesting that this mosquito is not a biologically competent vector [104]. Further studies are needed to evaluate whether cats are infected with *M. wenyonii* accidentally or more commonly in areas where large ungulates such cows and cats might have close contact.

*Anaplasma* and *Ehrlichia* spp. DNA was not detected in this study. A low PCR prevalence (1%) for these two tick-transmitted genera was previously reported in the Barcelona area [60]. In contrast, high molecular prevalence of *Ehrlichia canis* (9.9%) and *Anaplasma phagocytophilum* (8.4%) was described in cats from Madrid, Spain [29]. *Anaplasma* and *Ehrlichia* infections in cats are rare or sporadically documented worldwide [90, 105, 106], in agreement with the present study. Furthermore, *Babesia*, *Cytauxzoon*, and *Hepatozoon* DNA was also not detected in the present study. These tick-associated protozoan infections in cats appear to be rare, and have only been sporadically documented in cats from Mediterranean regions [107–109], as was found in the present study.

## Conclusion

In conclusion, *Bartonella* spp. and hemotropic *Mycoplasma* infections were found to be prevalent in cats residing in the Barcelona area, whereas no infection with *Anaplasma*, *Ehrlichia*, or Piroplasma species was detected by PCR testing. We report, for the first time, *B. koehlerae* infection in one apparently healthy cat in Spain. Co-infection with hemotropic *Mycoplasma* appears to be common in *Bartonella*-infected cats. Of note, *M. wenyonii* infection in cats is documented herein for the first time. Serological testing with the three *Bartonella* spp. antigens used in this study did not increase the overall seroprevalence as compared to IFA testing using only the *B. henselae* antigen. This and previous reports highlight the importance of combining serological and molecular diagnostic methods for the detection of *Bartonella* spp. infection. Future studies should focus on risk factors for acquiring co-infections with different *Bartonella* species and subspecies and whether co-infections might influence the clinical status and diagnosis of bartonellosis in cats. In addition, investigations are needed to better characterize the humoral immune response against *Bartonella* spp. to facilitate a better understanding of the immunological response to these bacteria in healthy and sick cats. Based upon our serology and PCR results, the risk of *Bartonella* and hemotropic *Mycoplasma* spp. transmission among cats, humans, and other animals in the Barcelona area may be substantial.

## Data Availability

The datasets supporting the conclusions of this article are included within the article. All analyzed data are available from the corresponding author upon reasonable request. Representative sequences generated in this study were submitted to the GenBank under accession numbers OK624784-OK624820.

## Abbreviations

Bp: Base pair
DNA: Deoxyribonucleic acid
EDTA: Ethylenediaminetetraacetic acid
ELISA: Enzyme-linked immunosorbent assay
IFA: Indirect immunofluorescence assay
ITS: Internal transcribed spacer
κ: Cohen’s kappa value
PBS: Phosphate-buffered saline
PCR: Polymerase chain reaction
cPCR: Conventional polymerase chain reaction
qPCR: Real time polymerase chain reaction
rRNA: Ribosomal ribonucleic acid
SE: Standard error
